# Acute myocarditis and rhabdomyositis after coronavirus disease 2019 messenger RNA vaccination and intensive training: a case report

**DOI:** 10.1186/s13256-026-05948-6

**Published:** 2026-03-13

**Authors:** Jieyu Wu, Attila Szakos, Bela Bozoky, Laszlo Szekely

**Affiliations:** 1 Department of Pathology/Cytology, Karolinska University Laboratory, 14186 Stockholm, Sweden; 2 Department of Pathology, The First Affiliated Hospital of Guangzhou Medical University, Guangzhou, 510230 China

**Keywords:** SARS-COV-2, Vaccination, Rhabdomyositis, Myocarditis, Case report

## Abstract

**Background:**

Despite the reporting of myocarditis associated with coronavirus disease 2019 vaccination, the correlation between vaccine-related rhabdomyositis and myocarditis incidence remains undocumented. This case offers a new perspective on myocarditis, potentially associated with the coronavirus disease 2019 vaccine, possibly caused by the strong immunostimulatory effects of messenger RNA vaccination-related rhabdomyositis.

**Case presentation:**

A 25-year-old male athlete of Swedish (white) ethnicity and non-Hispanic origin collapsed during a strenuous ice hockey training session after receiving his third dose of the coronavirus disease 2019 vaccine (BN162b2). He had received standard cardiopulmonary resuscitation, and the electrocardiography showed ventricle fibrillation. According to the laboratory test, elevations were observed in troponin T, aminotransferase, blood glucose, and leucocyte number. Magnetic resonance imaging indicated disseminated myocarditis. A biopsy of the heart revealed mild lymphocytic inflammation and focal myocytolysis. The patient had permanent brain damage and passed away after the breathing tube was removed. The autopsy revealed cardiac muscle necrosis and skeletal muscle rhabdomyolysis. There was a sign of massive expansion of lymphoid compartment in the gastrointestinal tract. Increased hemophagocytosis and eosinophils were seen in the lymph node and bone marrow. The specific case drew attention to a potential association between the coronavirus disease 2019 vaccine and fatal myocarditis.

**Conclusion:**

We suggest that strenuous training associated with skeletal muscle damage, together with the strong immunostimulatory effect of messenger RNA vaccination, may lead to autoimmune rhabdomyositis that potentially cross-reacts with the myocardium. Athletes should be aware of the potential danger of heavy training in close proximity to vaccination or acute infections.

## Introduction

The World Health Organization proclaimed that the coronavirus disease 2019 (COVID-19) pandemic was no longer a global health emergency in May 2023 after the worldwide spread of severe acute respiratory coronavirus 2 (SARS-CoV-2), which caused over 70,000 deaths from 2020 to 2023 [1]. However, the post-COVID era has continued to affect global health problems, including long-term post-COVID symptoms and post-vaccination effects [2]. Studies reported that patients who received the COVID-19 vaccination developed vaccination-related myocarditis [3], including with the most used messenger ribonucleic acid (mRNA)-based vaccines and vector vaccines. According to a study based on 2,000,287 individuals in the USA, 20 patients developed myocarditis after vaccination [4]. A systematic review article collected 24 studies with 37 cases and showed that different vaccinations could induce various myositis subtypes. Most patients had received Pfizer/BioNTech mRNA (BNT162b2) vaccination (56.7%) [5]. Though the occurrence of COVID-19 vaccination-related myocarditis is rare, it is important to note that potential vaccination-related myocarditis can have fatal consequences. In this study, we report on a young male athlete who trained hard after his third dose of COVID-19 vaccination and died owing to the consequences of acute myocarditis. Postmortem analysis of heart and skeletal muscle tissues showed an autoimmune reaction against cardiac and skeletal muscle fibers. The specific case drew attention to a potential association between the COVID-19 vaccine and fatal myocarditis by triggering an autoimmune reaction.

## Case presentation

A 25-year-old male athlete of Swedish (white) ethnicity and non-Hispanic origin reportedly vomited and collapsed on an outdoor training ground following several hours of intense physical exertion. First responders found the patient unconscious in cardiac arrest. Cardiopulmonary resuscitation had been administered for several minutes before the ambulance arrived. The electrocardiogram showed bradycardia, which progressed to ventricular tachycardia (VT). Electric cardioversion restored a normal pulse. In the emergency room, an initial blood test showed a low pH level (7.25) and a high troponin level (3760 ng/L). The pro-B-type natriuretic peptide (pro-BNP) was 60 ng/L (reference range < 84 ng/L). Drug tests were negative, which excluded drug-induced myocardial injury. Emergent computer tomography (CT) scans showed no detectable pathology in the skull and abdomen, except for infiltrates in the lung parenchyma. Echocardiogram revealed normal sinus rhythm and normal left ventricle size, and a significant decrease in left ventricular systolic function (around a 20% reduction). The right ventricle and valve had no detectable pathological changes. The primary diagnosis was cardiac arrest. Supportive treatment was administered to the patient, comprising glucose, sodium chloride, norepinephrine, tinzaparin, and the standard cardiac arrest protocol.

Medical history showed that the patient had received a third dose of the COVID-19 vaccine (BNT162b2) 2 weeks before. After getting vaccinated, he took a day off but continued to train intensively until he collapsed. A laboratory test showed an elevated procalcitonin level of 2.5 μg/L. The virus infection test, which included SARS-CoV-2, influenza A, and influenza B, showed no signs of infection. The level of SARS-CoV-2 spike protein exceeded 250 units/mL. Later, the patient exhibited chills and unconscious movements, accompanied by a fever of 37.8 °C. The patient was placed on a ventilator with an endotracheal tube, receiving a fraction of inspired oxygen (FiO_2_) of 0.3 and a positive end-expiratory pressure of 8 cm H_2_O. To ensure the safety of brain function, he was sedated using propofol, morphine, midazolam, and rocuronium. No signs of epilepsy were present, and the electroencephalogram (EEG) showed decreased brain activity.

In the intensive care unit, the patient showed stable respiratory function, with a partial pressure of carbon dioxide (pCO_2_) < 6 kPa, although plenty of yellow, foul-smelling mucus was found in the ventilator tube, and muffled sounds were heard in both lungs. Chills were continuous in the absence of elevated body temperature. No specific pathogens were identified from the tracheal and nasopharynx secretions. Subsequent laboratory testing showed high levels of C-reactive protein, creatine kinase, pro-brain natriuretic peptide (BNP), troponin T, serum neuro-specific enolase, and white blood cells (Table 1). Doppler echocardiography revealed a left ventricle of normal size and slightly impaired systolic function, with an ejection fraction between 45% and 50%, and hypokinesia in certain areas. The patient had a normal-sized right ventricle with normal systolic function and without signs of increased load, and normal-sized atria. A diagnosis of myocarditis was considered in possible relation to the vaccination. Other differential diagnoses, including infectious myocarditis, autoimmune/immune-mediated myocarditis, and myopericarditis were considered. Treatment with cortisone and cefotaxime was administered.

**Table 1:** The laboratory examination results of the patient on day 3 of hospitalization

Test	Result	Normal
C-reactive protein (CRP)	8.3 mg/dL	< 0.3 mg/dL
Creatine kinase	2540 IU/L	48–402 IU/L
Pro-brain natriuretic peptide, BNP	1210 ng/L	< 84 ng/L
Troponin T	1320 ng/L	< 15 ng/L
Serum neuro-specific enolase (NSE) White blood cell counts	53 μg/L 12.8 × 10^9^/L	< 17–20 μg/L 3.5–8.8 × 10^9^/L

**Fig. 1:**
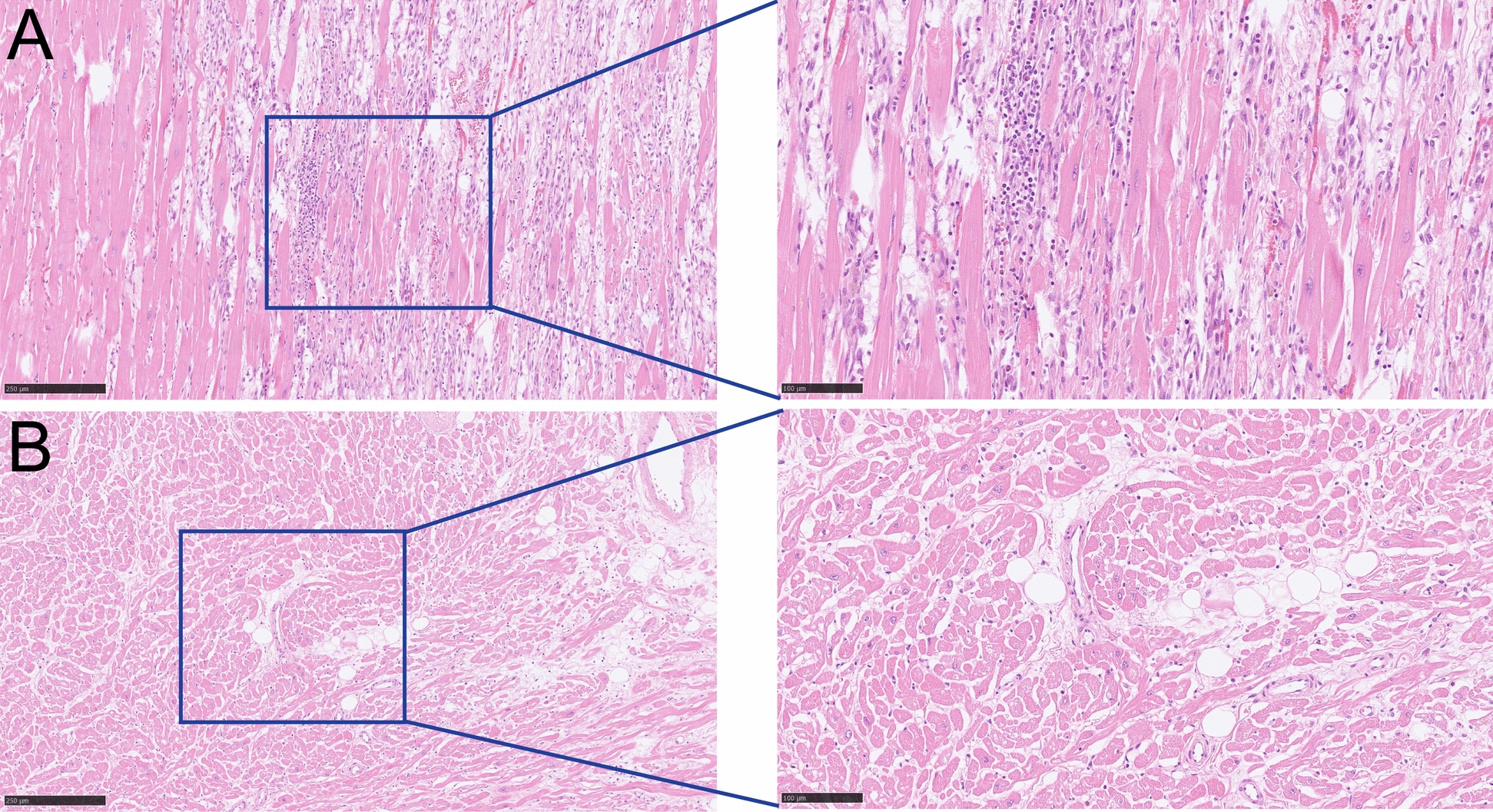
The histology of a heart biopsy. **A**. Focal lymphocytic inflammation of mild degree was observed within the cardiac mesenchyme. **B**. The cross-section showed myocytolysis with features of muscle cells with vacuolization, loss of cross-striations and pale cytoplasm (Magnificence 10X and 20X, scale bar = 250 μm and 100 μm)

**Fig. 2:**
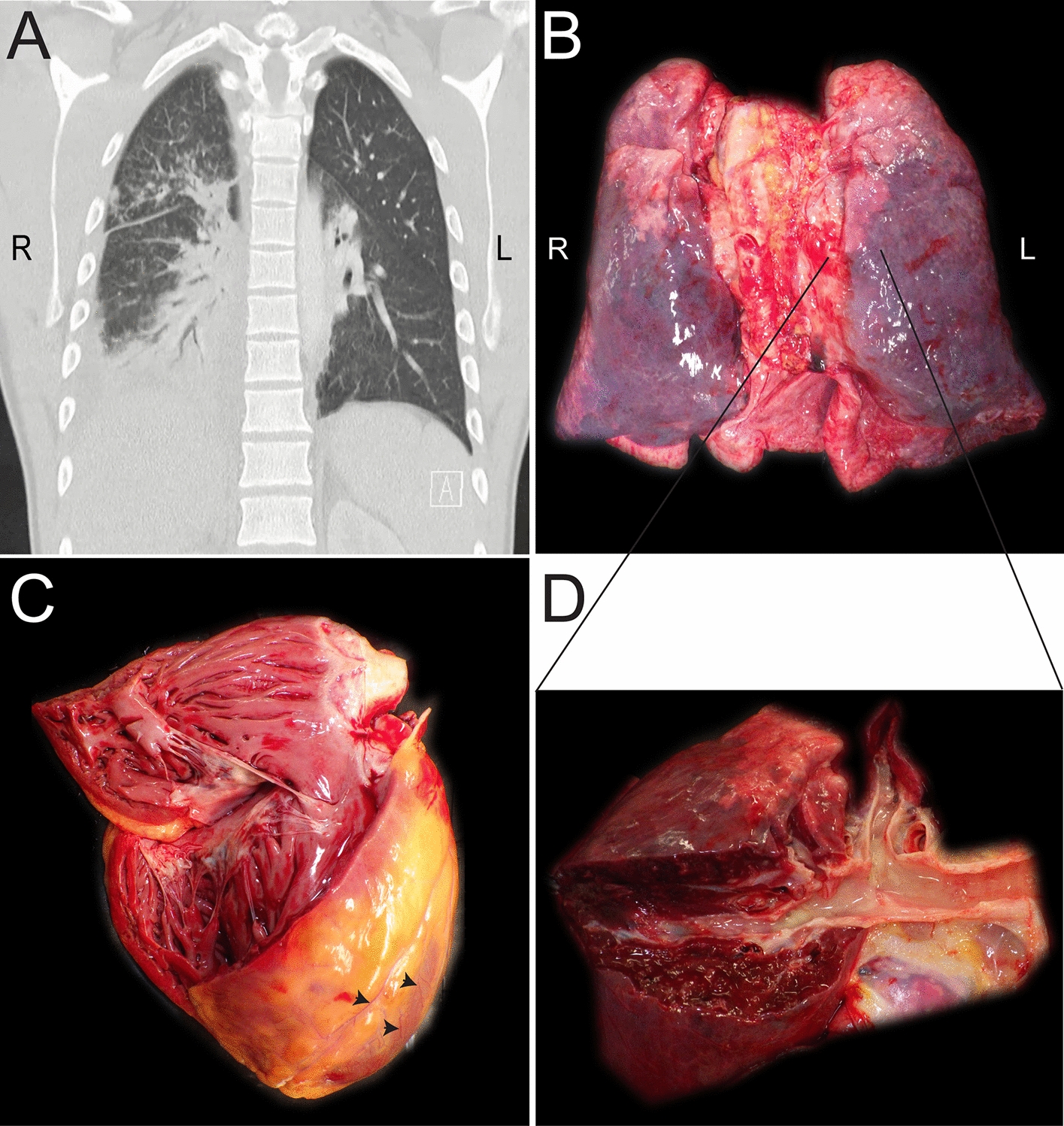
Images of chest computer tomography and gross examination of lung and heart. **A**. The representative image of the chest CT showed pneumonia with bilateral partially atelectatic lower lobes and peribronchial infiltration. **B**. Shiny and smooth surfaces of both lungs were demonstrated in the gross examination's representative image. **C**. The representative image of the heart's gross examination showed a reddish-brown hue with subtle grayish marbling, indicative of the myocardial tissue. An observable perivascular infiltration pattern was present around the small pericardial vessels under the visceral pericardium (arrow). **D**. The lung's longitudinal section exhibited viscous mucus, as well as a trachea and bronchi filled with purulence

Later, magnetic resonance imaging (MRI) revealed heart edema and fibrosis or necrosis as signs of myocarditis. It implied hypokinesia in the inferolateral, inferior, and apical part of the heart. The ejection fraction (EF) was 49% with a normal heart rate. Heart biopsy on day 5 after his admission showed signs of mild lymphocytic inflammation and focal myocytolysis. There was no eosinophil or giant cell infiltration and no amyloid deposition (Fig. 1). The EEG remained stable and showed no signs of epileptiform activity. The medical consilium decided to conduct a wake-up test on the following day. When the wake-up test was performed, the patient demonstrated intense shivering in the muscles. The body temperature and lactate acid level rose. He did not open his eyes and was coughing heavily. The EEG showed delta activity with a frequency of 1–2 Hz, but no epileptiform activity and no electrographic seizure activity. Considering the situation, the patient was sedated again. Later, a chest computed tomography (CT) scan showed pneumonia with bilateral partially atelectatic lower lobes and peribronchial infiltrates in both lower lobes and dorsally in the right upper lobe. There were no suspected tumors in airway tissue or infiltrates typical of COVID-19 (Fig. 2A). His repeated blood test showed a high CRP with a low white blood cell count. Although the neurologist suspected damage to both the supra- and infratentorial regions, the sustained EEG displayed background activity. The brain CT scan revealed the impact of ischemia/anoxia on both gray and white matter. Clonazepam was used to decrease the sedation level.

On day 6 after admission, the patient continued to have a strong cough with abundant thick yellow phlegm. A wake-up test was performed again while still on the ventilator. During the test, the patient coughed and trembled but remained still and unconscious. The physical check found his corneal reflex was normal on the right side but weak on the left side. Ciliary response was absent, with an atypical Doll’s eye maneuver when turning to the right. A permanent brain injury was assessed. Brain MRI revealed the presence of widespread diffusion restriction of ischemia within the bilateral occipital cortex, extending anteriorly in the bilateral media occipitotemporal gyrus. There was deep cytotoxic edema caused by ischemia in the bilateral caput nuclei caudate and the thalamus pulvinar. Thus, there were signs of hypoxic–ischemic damage in the supratentorial cortex bilaterally and in the basal ganglia and thalami. It was concluded that these brain injuries were permanent.

The doctors and the family had a breakpoint conversation and indicated that the overall prognosis was dismal. The patient would have a high probability of ending up in a vegetative state. On day 7 after admission, sedation was increased owing to muscle shivering and stretched movements in the arms. The family agreed to extubation in the afternoon, and soon after, the patient passed away. The suspected diagnosis was post-COVID-19 vaccination-associated myocarditis, which developed into  VT in connection with extensive training, and further led to cardiac arrest and subsequently developed into anoxic brain damage. To confirm the cause of death, the body was sent for autopsy.

The patient’s height was 180 cm, with a weight of 75 kg, resulting in a body mass index (BMI) of 23.1 kg/m^2^. The autopsy gross examination showed that both lungs had shiny and smooth surfaces (Fig. 2B). Viscous mucus and purulent exudate filled the trachea and bronchi. The cut surfaces of both lungs were grayish-red with moderate amounts of foamy fluid (Fig. 2D). With a weight of 422 g, the heart was slightly larger. The heart muscle presented with a reddish-brown color and a minor degree of grayish marbling. There was a noticeable perivascular infiltration pattern around small pericardial vessels under the visceral pericardium (Fig. 2C). The examination of the central nervous system revealed an absence of bleeding and thrombi in the dura and dural sinuses. However, the brain was swollen, and pressure grooves were seen on the cerebellar tonsils.

**Fig. 3:**
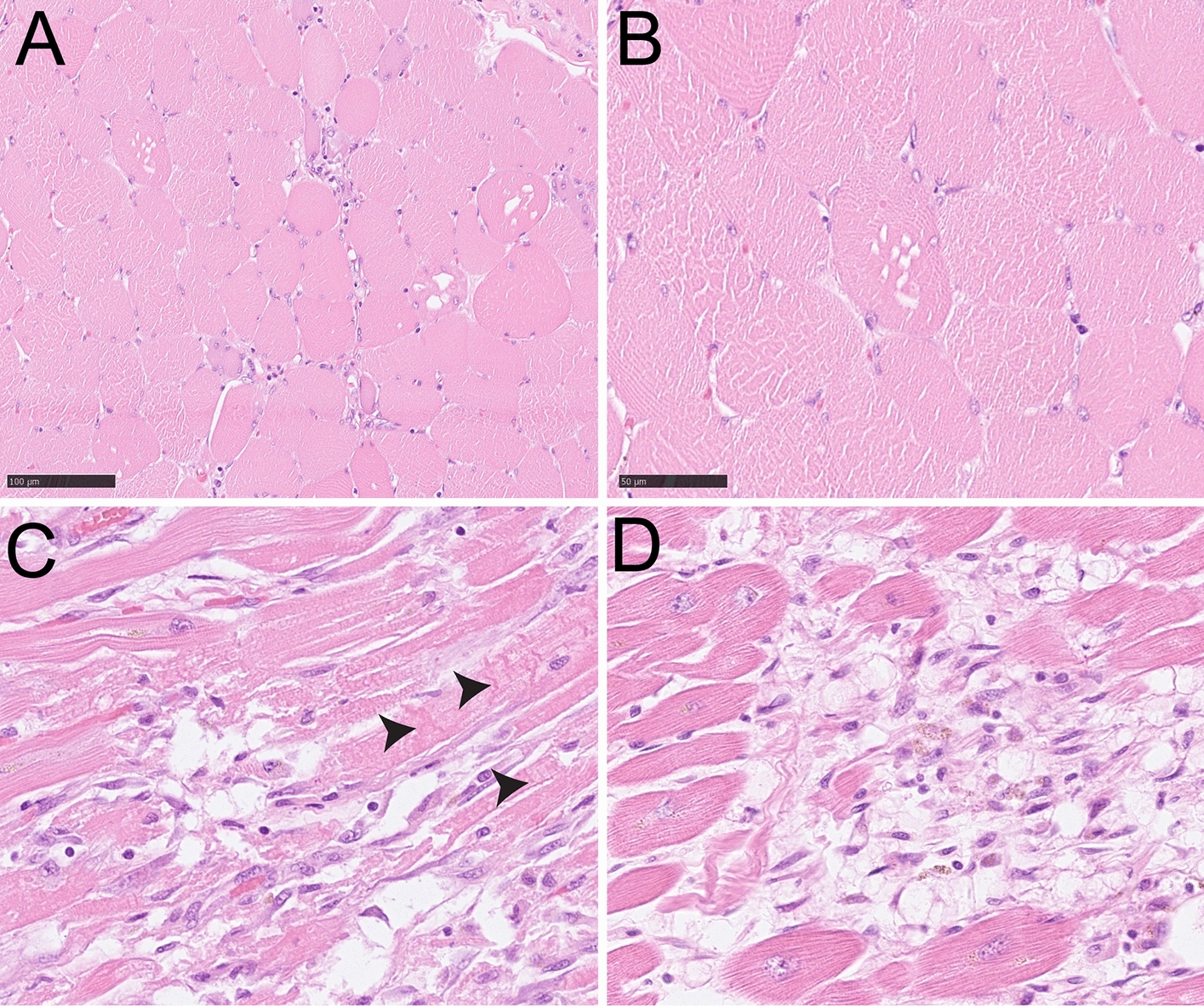
Histological features of skeleton muscle and cardiac muscle. **A**, **B**. Focal vacuolation was seen in the arm skeleton muscle. Mild inflammatory cells were observed infiltrating the mesenchyme. **C**. Contraction bands that represented a hypoxia-reperfusion injury and autoimmune effect were seen in the cardiac muscle (contraction bands were pointed by arrows). **D**. Focal hemosiderin deposition was detected in the mesenchyme of cardiac muscle

**Fig. 4:**
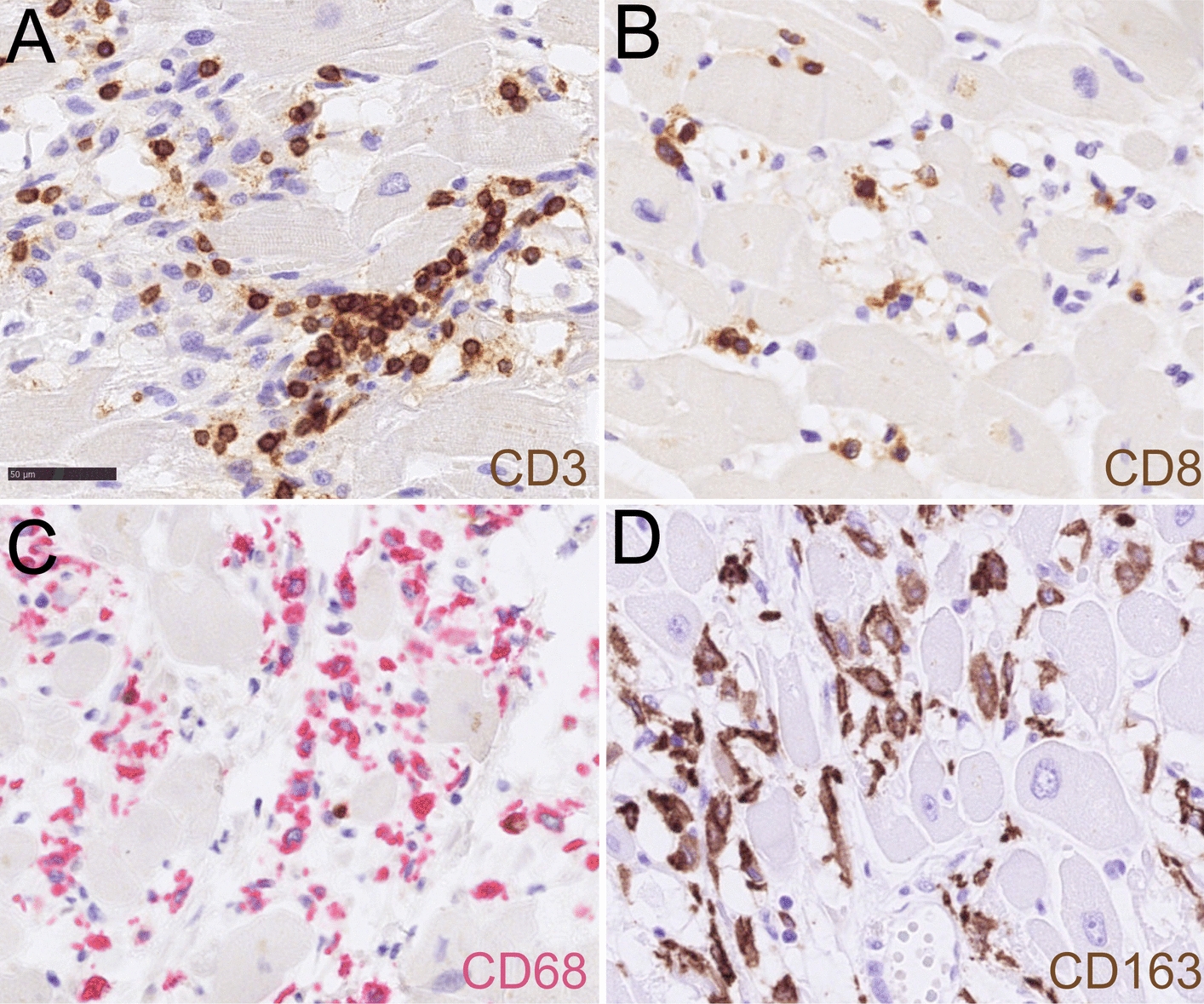
Immunohistochemistry staining of immune cells in the cardiac muscle. **A**-**D**. Focal immune cells infiltrated the mesenchyme of the cardiac tissue, showing a large amount of CD3 and CD8 positive lymphocytes and CD68 and CD163 positive macrophages

**Fig. 5:**
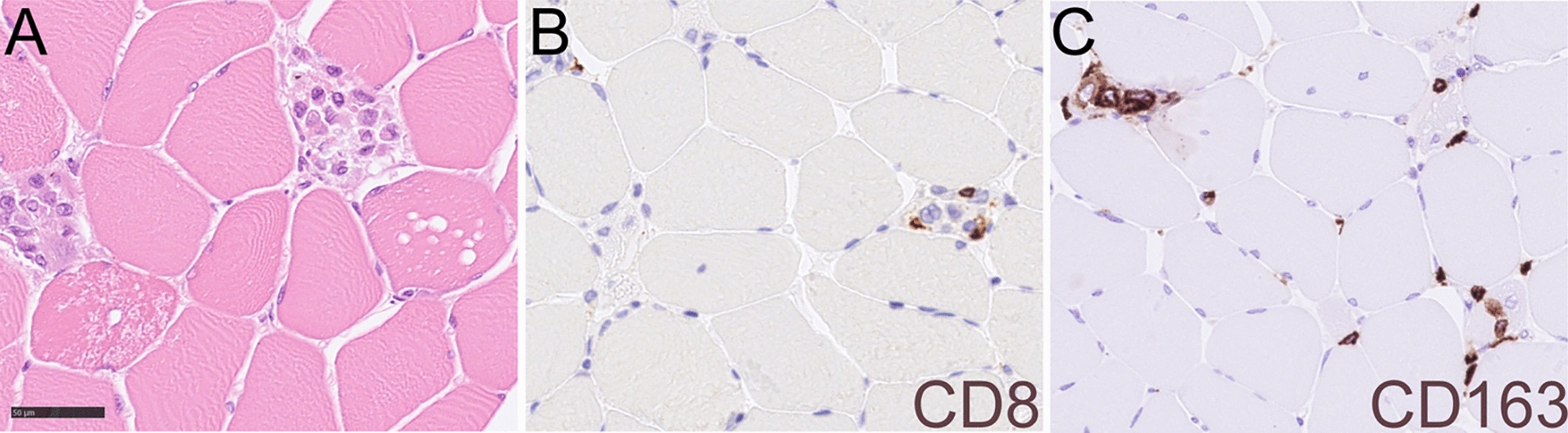
Histological features and immunohistochemical staining of skeleton muscle. **A**. Autopsy of skeleton muscle also found focal vacuolation and necrosis in the tissue. **B**, **C**. The immune cells infiltrated in the mesenchyme were mainly CD8-positive lymphocytes and CD163-positive macrophages

**Fig. 6:**
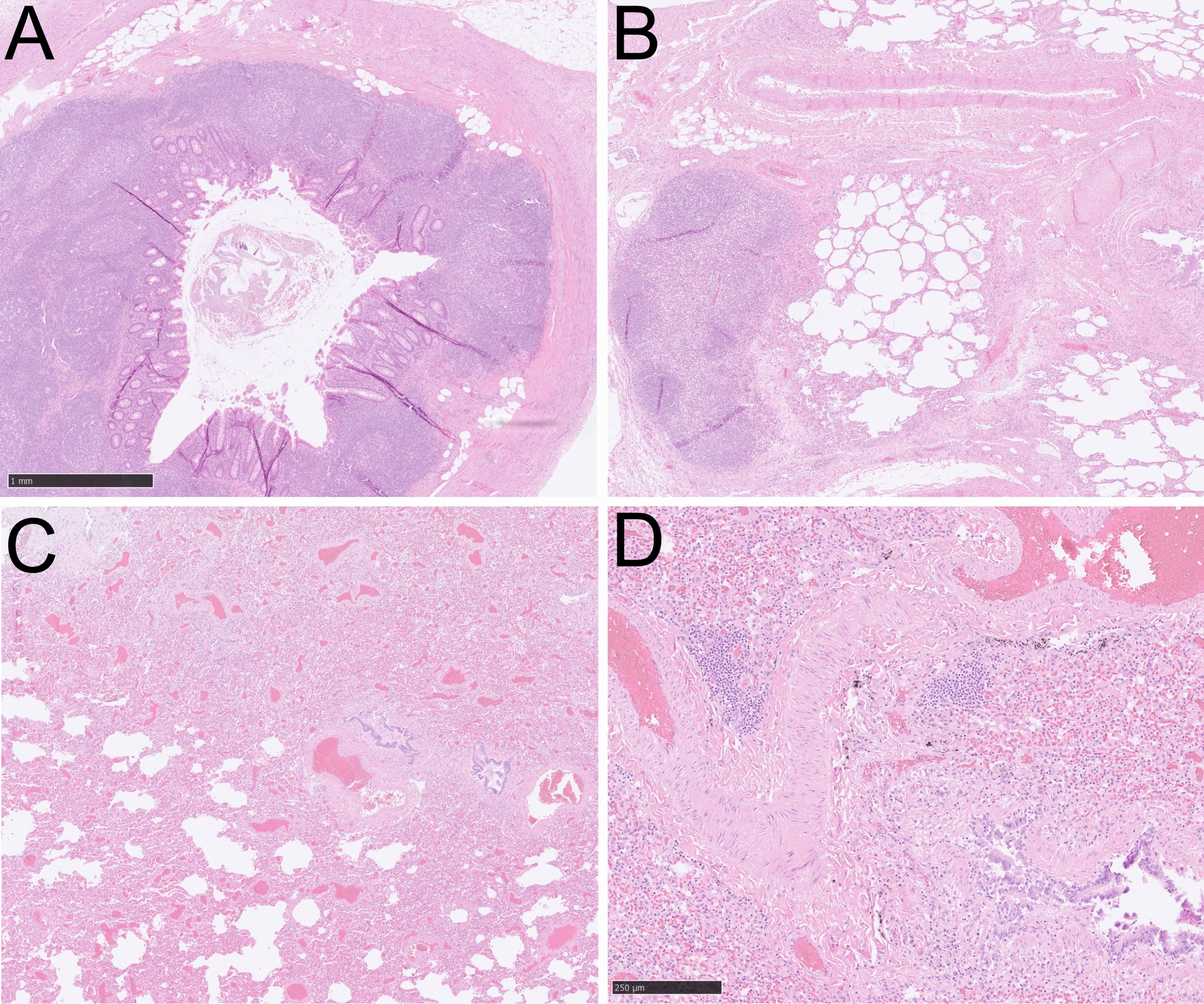
Histological characteristics of the gastrointestinal tract and lungs. **A**. Massive lymphoproliferation was seen in the lamina propria of the gastrointestinal tract. **B**. Focal lymphoproliferation was also seen in peribronchial of the lungs. **C**, **D**. There are increased amounts of macrophages in the lung alveolar stroma. Focal lymphoproliferation was observed in the lung tissue. (Magnificence 2.5X and 10X)

**Fig. 7:**
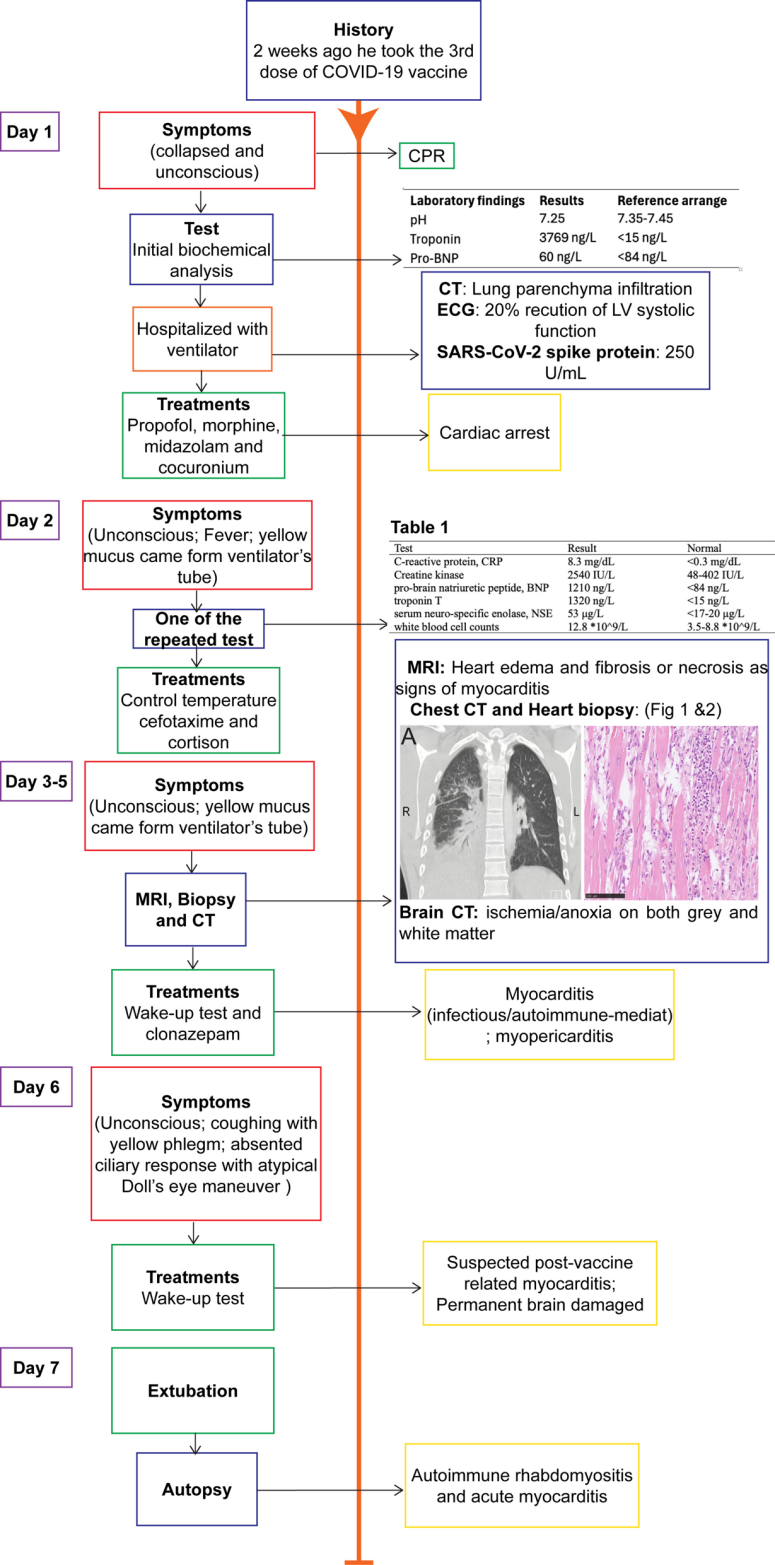
Timeline of the case. The schematic diagram of the timeline of the case (source: the authors own work). The patient demonstrated a rapid disease course. The final diagnosis was autoimmune rhabdomyositis and acute myocarditis, which was determined by the results of symptomatic, biochemical, radiographical and pathological examinations and autopsy results

Histological analysis of skeleton muscle found focal vacuolation and mild inflammatory cell infiltration in the mesenchyme (Fig. 3A,B). Focal necrosis, granulation tissue production, and vacuolating cardiomyocytolysis were seen in the heart muscle, forming as contraction band necrosis, and represented a hypoxia–reperfusion injury and autoimmune myocarditis (Fig. 3C,D). Immunohistochemistry showed a large amount of CD3- and CD8-positive lymphocytes, which were CD68- and CD163-positive (Fig. 4). In the muscle tissue, including the psoas, pectoralis, and intercostal muscles, focal immune cell-mediated rhabdomyolysis was seen. Most of the immune cells were CD8-positive T cells and CD163-positive macrophages (Fig. 5). There was massive activation of immune cells in the gastrointestinal tract, with the expansion of lymphoid follicles and accumulation of plasma cells and macrophages in the lamina propria of the ileum (Fig. 6A,B). In the lungs, there was a massive accumulation of macrophages in the alveoli, and focal lymphoproliferation was observed (Fig. 6C,D). In the lymphatic hematopoietic system, hemophagocytosis was seen in the lymph nodes and increased amounts of eosinophils in the bone marrow. The final diagnosis was autoimmune rhabdomyositis and acute myocarditis. It was triggered by the correlation of training-induced muscle damage and vaccination-induced muscle immune activation, which led to subsequent fatal autoimmune damage to the heart (Fig. 7).

## Discussion

Although the benefit–risk assessment of COVID-19 vaccination is favorable for all age and sex groups, and the incidence of myocarditis from COVID-19 vaccination is seven times lower than with SARS-CoV-2 infection [6], myocarditis has been reported as a complication post-COVID-19 messenger RNA (mRNA) vaccination, especially in young adult and adolescent male individuals [7–9]. In our case, we found that myocarditis coexisted with rhabdomyositis in the muscles of this patient, which pointed to a potential link between SARS-CoV-2 vaccination and the development of rhabdomyositis, followed by lethal myocarditis. The co-occurrence of myocarditis and rhabodmyositis had been mostly seen in patients with immune checkpoint inhibitors (ICIs) [10]. It was described in autoimmune diseases, for instance, antisynthetase syndrome, giant cell myocarditis, and COVID-19 infection, but is much less common outside ICI therapy [11–13].

The mechanism underlying the association of post-mRNA vaccination rhabdomyositis with subsequent fatal myocarditis remains incompletely understood. In a recent post-COVID-19 mRNA vaccine study, the authors found that post-vaccine-related acute myopericarditis was mediated by distinct immune components, including molecular mimicry, T cell receptor affinity, and homing imprinting to the heart. Data from both human and murine models revealed that the mRNA vaccine-encoded spike epitopes were homologous to cardiac self-proteins [14]. However, some other studies have demonstrated that mRNA vaccines do not need adjuvants owing to their strong specific immune stimulatory effect that is independent of the mRNA coded protein product [15, 16]. In our view, the most likely mechanism of SARS-CoV-2 vaccine that may have contributed to rhabdomyositis and subsequently myocarditis is the effect of the strong specific mRNA-induced immune stimulation that augmented the mechanical damage-induced inflammatory response and created an autoimmune response against the skeletal, and by cross-reaction, against the cardiac muscle. This interpretation differs from the proposed mechanism that anti-spike reactivity would be the main culprit behind the myocarditis through molecular mimicry [17]. Moreover, other previous studies have suggested that mRNA vaccines could transiently activate innate immune pathways, such as Toll-like receptors (*TLRs*), retinoid acid-inducible gene I (*RIG-I*), and melanoma differentiation-associated gene 5 (*MDA5*), leading to increased proinflammatory cytokines linked to autoimmune processes [18].

It has also been proposed that SARS-CoV-2 infection and released spike protein can also directly damage the myocardium of patients with COVID-19 [19]. We have previously shown that the SARS-CoV-2 virus was regularly absent in the heart tissues of COVID-19 victims and that the hearts suffered severe hypoxic damage as a consequence of diminished lung function [20]. Meanwhile, another study had found that both SARS-CoV-2 infection and mRNA vaccines increased c-MET-expressing T cells. However, the T cell reaction to cardiac muscle is weaker in those infected with SARS-CoV-2 compared with those vaccinated, suggesting different mechanisms for vaccine-induced and SARS-CoV-2-induced myocarditis [14]. From our current autopsy results, we found that the patient had marked inflammatory cell infiltration in most skeletal muscle tissues. Most of them were CD8-positive lymphocytes and CD163-positive macrophages, which caused muscle tissue damage and rhabdomyositis. The presence of lymphocytes in multiple muscle tissues may have initiated a systemic autoimmune response. This could have resulted in a cross-reaction with the myocardium, leading to myocarditis.

The proposed mechanism could lead to improved prevention of COVID-19, or any other mRNA vaccination-induced myocarditis. The restriction of intense physical activity subsequent to mRNA vaccination could potentially avert systemic autoimmune reactions prompted by muscular damage. This case also emphasizes the importance of histological and immunohistochemistry testing of multiple skeletal muscle samples from young athletes who die from myocarditis in relation to sporting events. Exercise restriction at a time of seemingly mild infections might also prevent some of the tragic sudden deaths of young athletes.

## Limitations

The present case has indicated a potential correlation between the COVID-19 mRNA vaccine and fetal myocarditis through the induction of an autoimmune response via rhabdomyolysis, and the limitations of this study should also be specified. It is a single case study that lacked a control group. The suggested pathophysiology of a cross-reaction autoimmune response is plausible but still hypothetical and lacks definitive proof. The hypotheses that we discussed merely offer a basis for understanding uncommon post-vaccination presentations and accentuate the necessity for further research into the underlying mechanisms. Additionally, our findings pertain specifically to the BNT162b2 mRNA vaccine and may not be generalizable to other mRNA vaccines.

## Conclusion

We have presented a case of a young athlete who died as a consequence of severe myocarditis following the combined events of COVID-19 mRNA vaccination and strenuous exercise. Our findings indicated that concomitant rhabdomyositis occurred, probably as a result of mechanical damage to skeletal muscle and potentially vaccine-associated immune system stimulation. We suggest a scenario that the lethal myocarditis was a secondary event due to the cross-reaction of the anti-skeletal muscle response against the heart muscle cells. We propose that a postmortem analysis of skeletal muscle samples be integrated into the examination of all lethal cases of myocarditis in athletes to identify possible rhabdomyositis. Additionally, we suggest that limiting exercise following vaccination or even common infections may prevent some instances of severe myocarditis.

## Methods and materials

### Tissue preparation and histology

The tissue preparation followed the laboratory procedure at Karolinska University Hospital, Huddinge. The tissues were formalin-fixed and paraffin-embedded. Sections were cut at 5 μm and stained with hematoxylin and eosin (H&E). Subsequently, all slides were examined under microscopy and scanned digitally using Nanozoomer S360 (Hamamatsu, Japan).

### Immunohistochemistry (IHC)

The IHC was conducted by Ventana Ultra Benchmark (Ventana Medical Systems, USA) and BOND-MAX (Leica Biosystem, Germany) according to the manufacturer’s instructions. CD3 (clone LN10 no. NCL-L-CD3-565, Novocastra), CD8 (clone C8/144B no. M7103, Dako, Agilent), CD163 (clone MRQ-26 no. 05973929001, Ventana), and CD68 (clone PG-M1 no. M0876, Dako, Agilent) were stained, respectively.

## Data Availability

Data and materials that support the findings of this study can be obtained from the corresponding author upon reasonable request.
